# Serum erythropoietin levels, breast cancer and breast cancer-initiating cells

**DOI:** 10.1186/s13058-019-1100-9

**Published:** 2019-01-30

**Authors:** Kruttika Bhat, Kiri Sandler, Sara Duhachek-Muggy, Claudia Alli, Fei Cheng, Neda A. Moatamed, Clara E. Magyar, Lin Du, Gang Li, Susan McCloskey, Erina Vlashi, Frank Pajonk

**Affiliations:** 1 Department of Radiation Oncology, David Geffen School of Medicine at UCLA, 10833 Le Conte Ave, Los Angeles, CA 90095-1714 USA; 2 Department of Pathology and Laboratory Medicine, David Geffen School of Medicine at UCLA, Los Angeles, CA USA; 3 Jonsson Comprehensive Cancer Center at UCLA, Los Angeles, CA USA; 4 Image Analysis/Virtual Microscopy, Translational Pathology Core Laboratory, Department of Pathology and Laboratory Medicine, Los Angeles, CA USA; 5 Department of Biostatistics, School of Public Health at UCLA, Los Angeles, CA USA

**Keywords:** Breast cancer-initiating cells, Erythropoietin, Radiation therapy

## Abstract

**Background:**

Cancer is frequently associated with tumor-related anemia, and many chemotherapeutic agents impair hematopoiesis, leading to impaired quality of life for affected patients. The use of erythropoiesis-stimulating agents has come under scrutiny after prospective clinical trials using recombinant erythropoietin to correct anemia reported increased incidence of thromboembolic events and cancer-related deaths. Furthermore, previous preclinical reports indicated expansion of the pool of breast cancer-initiating cells when erythropoietin was combined with ionizing radiation.

**Methods:**

Using four established breast cancer cell lines, we test the effects of recombinant human erythropoietin and the number of breast cancer-initiating cells in vitro and in vivo and study if recombinant human erythropoietin promotes the phenotype conversion of non-tumorigenic breast cancer cells into breast cancer-initiating cells. In a prospective study, we evaluate whether elevated endogenous serum erythropoietin levels correlate with increased numbers of tumor-initiating cells in a cohort of breast cancer patients who were scheduled to undergo radiation treatment.

**Results:**

Our results indicate that recombinant erythropoietin increased the number of tumor-initiating cells in established breast cancer lines in vitro. Irradiation of breast cancer xenografts caused a phenotype conversion of non-stem breast cancer cells into induced breast cancer-initiating cells. This effect coincided with re-expression of the pluripotency factors c-Myc, Sox2, and Oct4 and was enhanced by recombinant erythropoietin. Hemoglobin levels were inversely correlated with serum erythropoietin levels, and the latter were correlated with disease stage. However, tumor sections revealed a negative correlation between serum erythropoietin levels and the number of ALDH1A3-positive cells, a marker for breast cancer-initiating cells.

**Conclusions:**

We conclude that physiologically slow-rising serum erythropoietin levels in response to tumor-related or chemotherapy-induced anemia, as opposed to large doses of recombinant erythropoietin, do not increase the pool of breast cancer-initiating cells.

**Electronic supplementary material:**

The online version of this article (10.1186/s13058-019-1100-9) contains supplementary material, which is available to authorized users.

## Background

Radiation therapy (RT) is an integral part of the multi-modality treatment paradigm for breast cancer, as it improves loco-regional control as well as breast cancer-related mortality [1]. The majority of patients experience durable local control after radiotherapy for breast cancer. However, for those few who do relapse locally, the impetus is often unknown.

A large percentage of patients with breast cancer require chemotherapy as part of their treatment, which is known to impair normal hematopoiesis, causing anemia. Additionally, a substantial number of even early-stage breast cancer patients suffer from tumor-related anemia at the start of the treatment regimen [2] as cancers often induce myelosuppressive cytokine profiles [3]. The resulting low pre-treatment hemoglobin levels are correlated with an unfavorable prognosis [2]. The underlying mechanisms are incompletely understood, but low hemoglobin levels trigger the secretion of erythropoietin [4], which has been shown to increase the number of radiation and chemotherapy-resistant tumor-initiating cells in breast cancer [5, 6] and glioma [7]. A substantial body of evidence supports the view that erythropoietin is secreted within tumors in a para- and autocrine fashion [7–9].

Recombinant human erythropoietin (rhEpo) has been widely used in patients suffering from tumor-related or chemotherapy-induced anemia to improve quality of life (QOL) and to increase loco-regional tumor control after RT [10]. Initially, rhEpo was FDA-approved for the treatment of chemotherapy-induced anemia in cancer patients. Studies designed to investigate the safety profile of rhEpo in cancer patients did not indicate non-hematological targets of Epo in vitro or in vivo.

While the vast majority of placebo-controlled prospective clinical trials were designed to address the impact of rhEpo on QOL, a few studies were designed with loco-regional control or overall survival as primary endpoints. Unexpectedly, besides reporting an increased incidence of cardio-vascular events, the latter studies revealed impaired tumor control in patients treated with rhEpo [11–13]. Additionally, a recent meta-analysis confirmed increased mortality for cancer patients treated with rhEpo [10]. The use of rhEpo in cancer patients is now restricted to palliative settings.

Together these findings led us to hypothesize that elevated endogenous erythropoietin levels could similarly increase the aggressiveness of breast cancer and potentially impair treatment outcomes. In the present study, we demonstrate that in a cohort of breast cancer patients, while endogenous erythropoietin levels were negatively correlated with hemoglobin levels, higher erythropoietin levels correlated with lower numbers of putative breast cancer-initiating cells (BCICs) in the tumors.

## Methods

### Cell culture

Human SUM159PT breast cancer cell lines were purchased from Asterand (Asterand, Inc., MI). Human MDA-MB-231, MCF-7, and T47D breast cancer cell lines were purchased from American Type Culture Collection (Manassas, VA). SUM159PT-ZsGreen-cODC, MDA-MB-231-ZsGreen-cODC, MCF-7-ZsGreen-cODC, and T47D-ZsGreen-cODC cell lines were obtained as described previously [14]. SUM159PT cells were cultured in log-growth phase in F12 Medium (Invitrogen, Carlsbad, CA) supplemented with 5% fetal bovine serum (FBS; Sigma Aldrich, St Louis, MO), penicillin (100 units/ml) and streptomycin (100 μg/ml) (both Invitrogen), and insulin (5 μg/mL) and hydrocortisone (1 μg/ml). MCF-7 cells were cultured in MEM media supplemented with non-essential amino acids, sodium pyruvate, insulin, penicillin, streptomycin, and 10% FBS. MDA-MB-231 and T47D cells were cultured in log-growth phase in Dulbecco’s modified Eagle’s medium (DMEM) (Invitrogen) supplemented with 10% fetal bovine serum, penicillin, and streptomycin. All cells were grown in a humidified incubator at 37 °C with 5% CO_2_. Cell line identities were confirmed by DNA fingerprinting (Laragen, Culver City, CA).

### Animals

Six- to 8-week-old NOD-*scid* IL2Rgamma^null^ (NSG) mice, originally obtained from The Jackson Laboratories (Bar Harbor, ME), were re-derived, bred, and maintained in a pathogen-free environment in the American Association of Laboratory Animal Care-accredited Animal Facilities of the Department of Radiation Oncology, University of California (Los Angeles, CA), in accordance to all local and national guidelines for the care of animals. Weight of the animals was recorded every 2 days.

### Flow cytometry

Mammospheres were harvested, dissociated using TrypLE (Thermo Fisher Scientific) and fire-polished Pasteur pipettes, and passed through a 70-μm sieve (Millipore). Explanted tumors were digested using a GentleMACS dissociator (Miltenyi Biosciences, Auburn, CA). Single-cell suspensions were subjected to flow cytometry (MACSQuant Analyzer, Miltenyi), and ZsGreen and/or BFP expression was analyzed using the FlowJo software package (v10, FlowJo, Ashland, OR).

BCICs and non-stem breast cancer cells were isolated based on the expression of our reporter construct using high-speed FACS.

### In vitro sphere formation assay

In order to assess self-renewal capacity, cells were trypsinized and plated in mammosphere media (DMEM-F12, 0.4% BSA (Sigma), 10 ml/500 ml B27 (Invitrogen) 5 μg/ml bovine insulin (Sigma), 4 μg/ml heparin (Sigma), 20 ng/ml fibroblast growth factor 2 (bFGF, Sigma), and 20 ng/ml epidermal growth factor (EGF, Sigma)) into 96-well ultra-low adhesion plates, ranging from 1 to 256 cells/well. Growth factors, EGF and bFGF, were added every 3 days, and the cells were allowed to form mammospheres for 20 days. The number of spheres formed per well was then counted and expressed as a percentage of the initial number of cells plated.

### Quantitative reverse transcription-PCR

Total RNA was isolated using TRIZOL Reagent (Invitrogen). cDNA synthesis was carried out using the SuperScript Reverse Transcription III (Invitrogen). Quantitative PCR was performed in the My iQ thermal cycler (Bio-Rad, Hercules, CA) using the 2× iQ SYBR Green Supermix (Bio-Rad). *C*_t_ for each gene was determined after normalization to PPIA, TBP, and IPO8, and ΔΔ*C*_t_ was calculated relative to the designated reference sample. Gene expression values were then set equal to 2^−ΔΔCt^ as described by the manufacturer of the kit (Applied Biosystems). All PCR primers were synthesized by Invitrogen and designed for the human sequences of Oct4, Sox2, Nanog, Klf4, c-Myc, and the housekeeping genes PPIA, TBP, and IPO8. Primer sequences are listed in an additional file (see Additional file 1).

### In vivo limiting dilution assay

High-speed FACS based on the accumulation of the ZsGreen reporter protein was used to sort SUM159PT-ZsGreen-cODC breast cancer cells. 10^2^–10^6^ ZsGreen-positive or ZsGreen-negative cells were injected s.c. into the flanks of 6–8-week-old NSG mice. Tumor formation was monitored for 16 weeks after injection. Frequencies of tumor-initiating cells were calculated as described in [15].

### Irradiation

Cells were irradiated at room temperature using an experimental X-ray irradiator (Gulmay Medical Inc. Atlanta, GA) at a dose rate of 5.519 Gy/min for the time required to apply a prescribed dose. The X-ray beam was operated at 300 kV and hardened using a 4-mm Be, a 3-mm Al, and a 1.5-mm Cu filter. Corresponding controls were sham irradiated.

For tumor irradiation studies, the animals were anesthetized with isofluorane. Tumors growing subcutaneously were localized in cone beam CT images, contoured in the SmART Plan software package and irradiated using a single beam from an image-guided small animal irradiator with a 10-mm circular collimator. The X-ray beam was operated at 225 KV (SmART, Precision X-Ray).

NIST-traceable dosimetry was performed on both X-ray machines.

### In vivo drug treatment

In order to test the effect of erythropoietin on radiation-induced reprogramming in vivo SUM159PT cells were stably infected with lenti-viral vectors coding for ZsGreen-cODC or BFP/ZsGreen-cODC. ZsGreen-cODC-positive intrinsic tumor-initiating cells were co-injected with BFP-positive/ZsGreen-cODC-negative, non-tumorigenic cells. When tumors reached 0.5 cm in diameter, a single radiation dose of 4 Gy was applied to the tumor. From the day of radiation treatment erythropoietin was administered for five consecutive days at 500 IU/kg i.p. (AMGEN, Thousand Oaks, CA), starting 3 h before irradiation of the tumors. Control animals received a single radiation dose of 4 Gy to the tumor and/or saline injections. Twenty-four hours after the final application of erythropoietin mice were euthanized and the tumors explanted. Explanted tumors were analyzed for the induction of BCICs and stained for Klf4 expression using an anti-Klf4 antibody (Abcam, ab215036, 1:2000 dilution), and Sox2 expression using an anti-Sox2 antibody (Cell Signaling Technology, 14962S, 1:50 dilution) overnight at 4 °C. The slides were digitized on a ScanScope AT (Aperio Technologies, Inc., Vista, CA) and morphimetric analysis performed with *Definiens’* Tissue Studio (Definiens Inc., Parsippany, NJ) to determine the percentage of Klf4- and Sox2-positive cells in a non-biased method. Briefly, using the pre-defined nuclear detection module and classification tool, positive and negative nuclei within each tissue cross section were identified. Thresholds were set to classify hematoxylin stain for negative nuclei and 3,3′-diaminobenzidine (DAB) stain for positive nuclei. The data were exported to GraphPad Prism for further statistical analysis.

Scanning and analysis were performed through the Translational Pathology Core Laboratory, Department of Pathology and Laboratory Medicine, David Geffen School of Medicine at UCLA.

### Patients

Female breast cancer patients treated between December 2012 and July 2016 at the Department of Radiation Oncology at the University of California, Los Angeles, were included in this study. The study was approved by the local ethics committee and performed in accordance with the revised Declaration of Helsinki. Serum samples were obtained before the start of radiation treatment. None of the patients received erythropoietin medication before the blood sampling and start of radiation treatment. Complete follow-up data were available through May 2017. As of May 2017, 96/99 patients were disease-free and alive.

### Erythropoietin enzyme-linked immunosorbent assays

Enzyme-linked immunosorbent assays (ELISA) were performed by following the manufacturer’s instructions (Human Erythropoietin Quantikine IVD ELISA Kit, R&D Systems, Minneapolis, MN). Briefly, 100 μL of erythropoietin (Epo) assay diluent was added to each well of the human erythropoietin-specific ELISA strip. One hundred microliters of standard, control, or serum samples from patients was added to each well and incubated for 2 h at room temperature (RT). The unknown serum samples were run in duplicates. The wells were thoroughly aspirated, and 200 μL of erythropoietin conjugate was added to each well and incubated for 2 h at RT. After washing for four times, 200 μL of substrate solution was added to each well. Plates were incubated at RT for 20–25 min after which the reaction was quenched by adding 100 μL of stop solution. The absorbance was read at 450 nm (Spectramax M5, Molecular Devices, Sunnyvale, CA). A wavelength correction was performed by subtracting readings at 600 nm from the readings at 450 nm. A standard curve was generated (GraphPad Prism, version 6), and the concentrations of erythropoietin in each serum sample were calculated by plotting the unknown O.D. values against the standard.

### Tissue microarrays

Tissue microarrays were constructed by the UCLA Translational Pathology Core Laboratory using a Veridiam VTA-110CC Semiautomated Tissue Arrayer using standard protocols. Tissue microarrays were stained against ALDH1A3 using a rabbit anti-human ALDH1A3 antibody (Thermo Fisher Scientific, PA5-29188) at the dilution of 1:1000 overnight at 4 °C, and against PSMD1 antibody (Abcam, ab2941) at the dilution of 1:500 overnight at 4 °C. The slides were rinsed with PBST and were incubated with Dako EnVision+ System- HRP Labeled Polymer Anti-Rabbit (Dako, K4003) at room temperature for 30 min. After a rinse with PBST, the slides were incubated with DAB (3,3′-diaminobenzidine) for visualization. Subsequently, the slides were washed in tap water, counterstained with Harris’ Hematoxylin, dehydrated in ethanol, and mounted with media. Tumor cores were scored by a board-certified breast cancer pathologist based on ALDH1A3 expression on membranes and cytoplasm or cytoplasmic PSMD1 expression on a scale from 0 to + 3 (0: no reaction, + 1 reaction barely visible, + 2 reaction clearly visible, + 3 reaction intense). ALDH1A1 expression was scored by the same principle.

### Statistical methods

Unless indicated otherwise, all data result from at least three biologically independent experiments. A *p* value of 0.05 in Student’s *t* test or ANOVA (SAS JMP, version 13) was considered an indicator for statistical significance.

For analyzing the clinical data, descriptive statistics such as mean and standard deviation were used to summarize continuous variables, while count and percentage were used for categorical variables. Student’s *t* test or univariate logistic regression was performed to evaluate the association between serum Epo levels vs PSMD1 expression and Epo vs ALDH1A3 expression. A multivariable linear regression model for Epo was constructed by including PSMD1 (yes (score 2 or 3) vs no (score 0 or 1)), and ALDH1A3 (yes (score 2 or 3) vs no (score 0 or 1)), age, cancer stage, Hb, triple-negative (yes vs no), chemo (yes vs no), receptor (positive vs negative), and histology. Model diagnostics were performed to assess the final multivariate regression model. For all statistical investigations, tests for significance were two-tailed, with a statistically significant *p* value threshold of 0.05. Statistical analyses were carried out using SAS version 9.2 (SAS Institute Inc., Cary, NC).

## Results

### Breast cancer cells with low 26S proteasome activity have increased tumorigenicity

We had previously demonstrated that BCICs downregulate proteasome subunit expression and function via binding of Musashi1 to the mRNA of NF-YA [16], a component of NF-Y, the master regulator of proteasome subunit expression [17]. Furthermore, we developed an imaging system that allows for tracking and targeting of BCICs based on the accumulation of a fusion protein of the fluorescent protein ZsGreen and the C-terminal degron of murine ornithine decarboxylase. In cells with low proteasome activity, the fusion protein cannot be degraded and allows for identifying these cells by flow cytometry [14, 18]. Importantly, breast cancer cells with low proteasome activity overlap with cells that exhibit high ALDH1 activity [19]. To demonstrate that SUM159PT breast cancer cells with low proteasome activity are enriched for BCICs, we performed an in vivo limiting dilution assay. 10^2^–10^6^ cells with high (ZsGreen-negative) or low (ZsGreen-positive) proteasome activity were injected into the flanks of female NSG mice. Tumor-initiating cell frequency was found to be 1:2586 in ZsGreen-positive cells and 1:72,886 in ZsGreen-negative cells (Table 1, *p* < 0.0001). This level of BCIC enrichment compared well to other used markers in the field [20], thus supporting the validity of our reporter system as a marker for BCICs in this triple-negative breast cancer line.

**Table 1:**
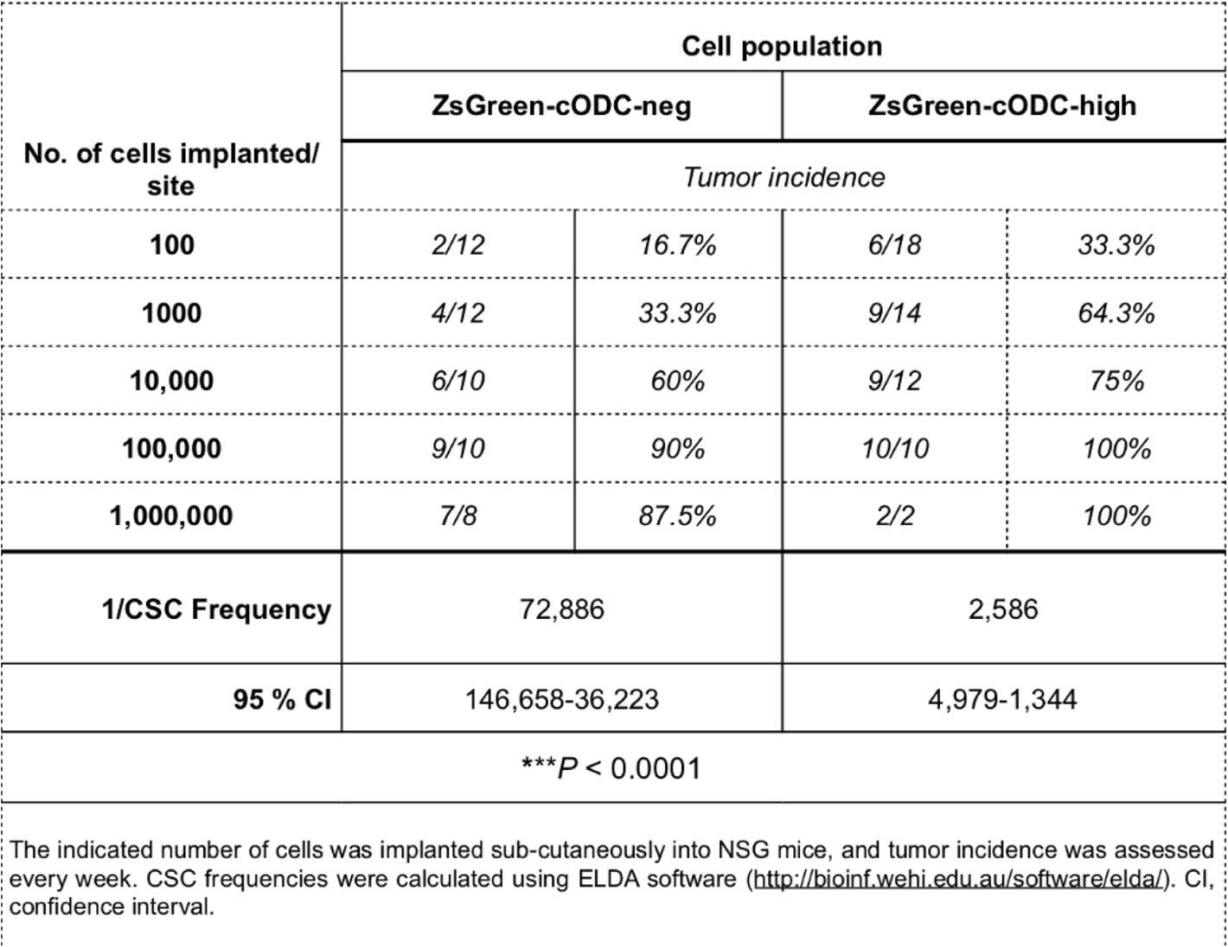
In vivo limiting dilution assay with SUM159-ZsGreen-cODC cells

### Radiation-induced enrichment of tumor-initiating cells and erythropoietin

**Fig. 1:**
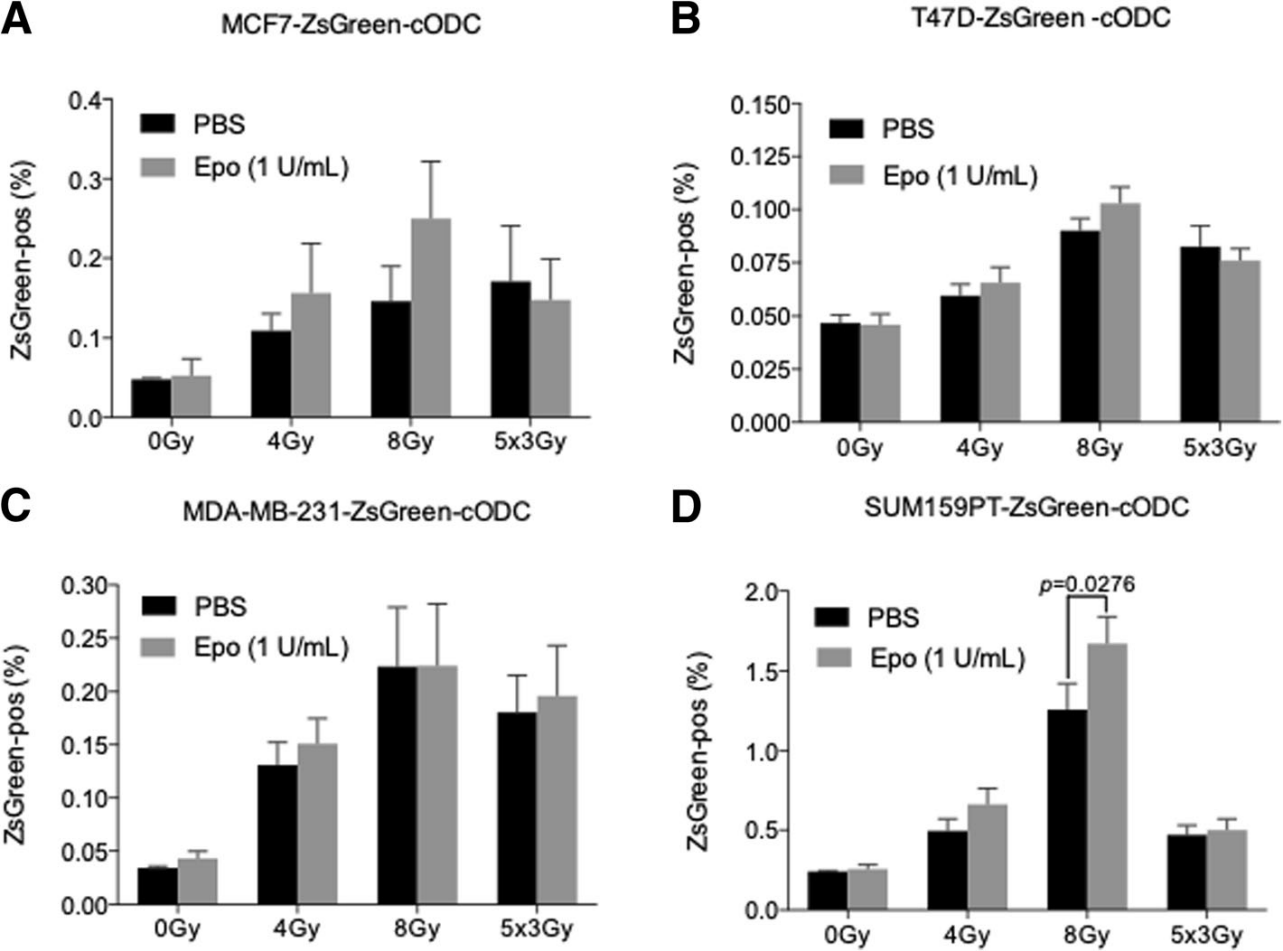
rhEpo enriches for the intrinsic BCICs *in vitro.* **a** MCF7-ZsGreen-cODC, **b** T47D-ZsGreen-cODC, and **c** MDA-MB-231-ZsGreen-cODC were plated into sphere cultures. **d** SUM150PT-ZsGreen-cODC were plated in adherent conditions. **a**–**d** The cells were treated with 1 IU/ml rhEpo (gray bars) or vehicle (black bars) and 1 h later were subjected to 0, 4, and 8 Gy or five daily doses of 3 Gy. Six days after Epo treatment, the cells were harvested and analyzed by flow cytometry. The graph represents the average %ZsGreen-positive BCICs from at least three independent repeats ± SEM. * *p* < 0.05, ** *p* < 0.01

In our previous studies, we found that radiation selects for radiation-resistant tumor-initiating cells in breast cancer [21, 22] and others have confirmed our findings [23]. Furthermore, we reported that erythropoietin increased the number of CD24^−/low^/CD44^high^ cells in vitro and increased mammosphere formation [5]. This marker combination was the first profile identified to enrich for BCICs [24]. When we treated mammospheres of MCF-7, T47D, MDA-MB-231, or SUM159PT cells with erythropoietin (1 IU/ml) and/or 0, 2, 4, 6, and 8 Gy or 5 × 3 Gy of radiation, we observed a significant radiation dose-dependent increase in the number of ZsGreen-positive BCICs (Fig. 1a–d), which was in agreement with our previous reports [21, 22]. However, addition of erythropoietin enhanced this effect only in Sum159PT cells and only after 8 Gy (Fig. 1d).

### Radiation-induced phenotype conversion of non-tumorigenic cells and erythropoietin

Increased numbers of BCICs observed after radiation treatment are not solely a result of their intrinsic relative radioresistance [21, 22] and selective killing of non-tumorigenic cells but also caused by radiation-induced phenotype conversion of non-tumorigenic breast cancer cells into BCICs [19]. This radiation-induced phenotype conversion can be reduced by inhibiting the PI3K and Notch pathways [5, 19]. Because erythropoietin is known to activate both the PI3K [25] and Notch signaling pathways [5], we sought to test if erythropoietin also promotes radiation-induced phenotype conversion.

**Fig. 2:**
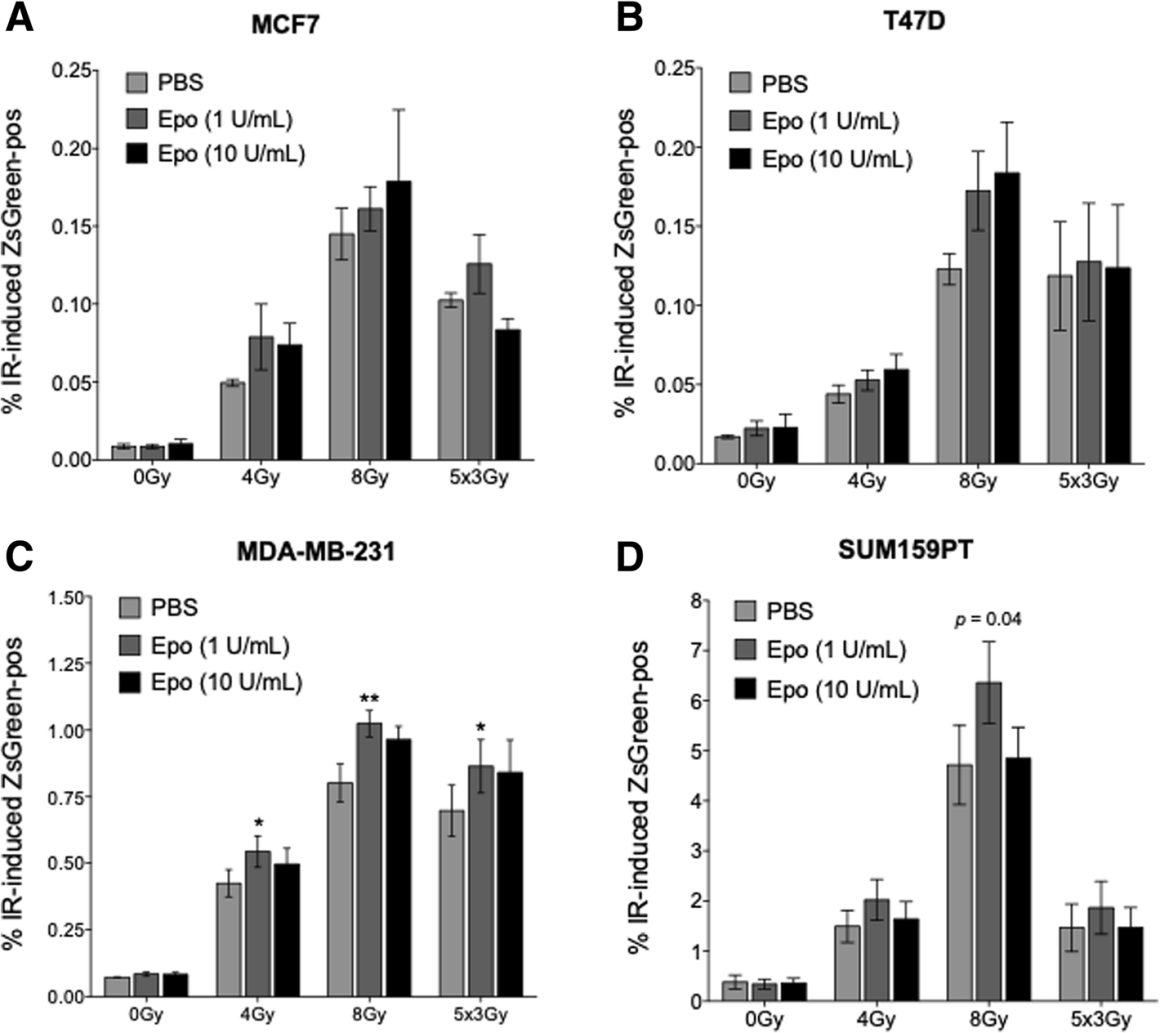
rhEpo enhances radiation-induced phenotype conversion in vitro*.* **a** MCF7-ZsGreen-cODC, **b** T47D-ZsGreen-cODC, **c** MDA-MB-231-ZsGreen-cODC, and **d** SUM150PT-ZsGreen-cODC were depleted of intrinsic BCICs by sorting for ZsGreen-negative cells. The cells were plated and the following day were treated with vehicle (light gray), 1 IU/ml rhEpo (dark gray), or 10 IU/ml rhEpo (black). One hour later, the cells were subjected to 0, 4, and 8 Gy or five daily doses of 3 Gy. Six days after rhEpo treatment, the cells were harvested and analyzed by flow cytometry. Graph represents the average %ZsGreen-positive cells from at least four independent repeats ± SEM. Statistical significance was determined by Student’s *t* tests. * *p* < 0.05, ** *p* < 0.01

In order to remove intrinsic BCICs, ZsGreen-cODC-expressing breast cancer cells were depleted from ZsGreen-positive BCICs using FACS. The remaining ZsGreen-negative non-stem breast cancer cells were irradiated with 0, 2, 4, 6, and 8 Gy or 5 × 3 Gy and treated with 0 or 1 IU/ml of rhEpo. Five days after radiation treatment, the number of induced BCICs was assessed by flow cytometry. Single radiation doses led to a dose-dependent increase in the number of induced BCICs, and this effect was most pronounced in triple-negative MDA-MB-231 and SUM159PT cells. Five daily doses of 3 Gy also induced the formation of BCICs, and both findings were in agreement with our previously published data [19]. The addition of rhEpo enhanced this effect (Fig. 2).

**Fig. 3:**
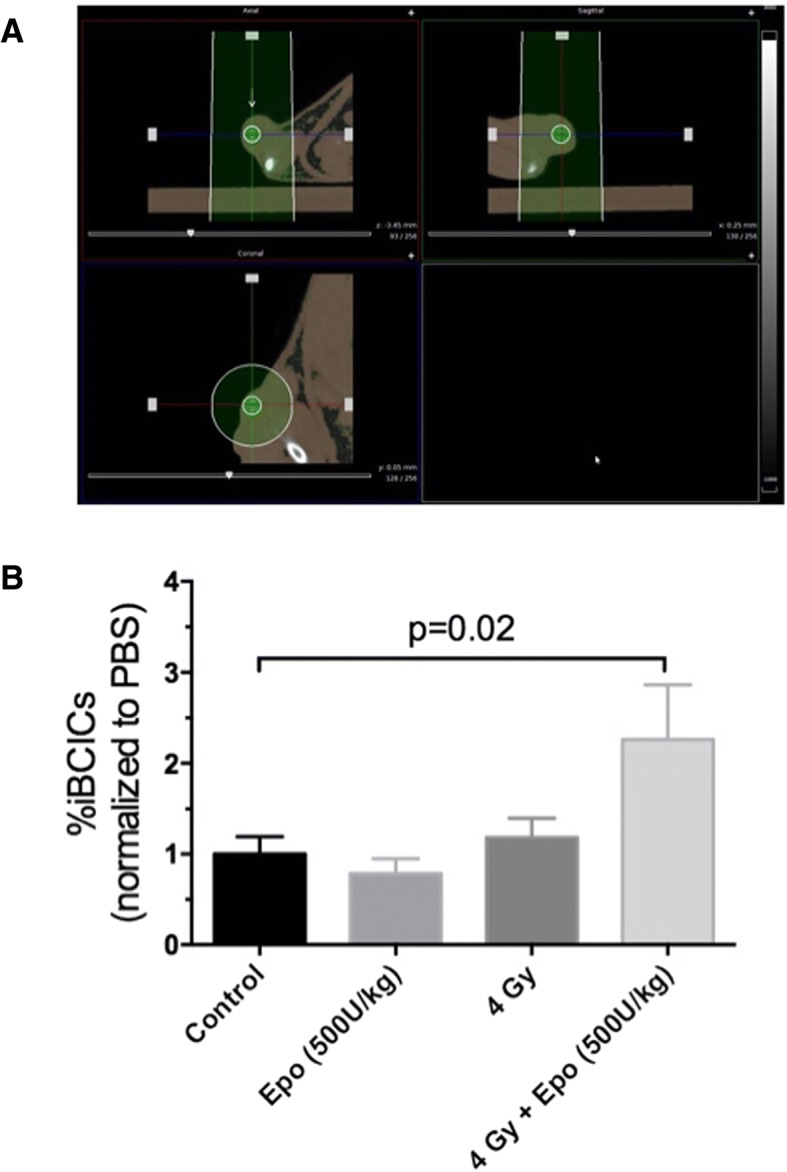
rhEpo enhances radiation-induced phenotype conversion in vivo. SUM159PT-StrawberryRed-ZsGreen-cODC cells were sorted for StrawberryRed-positive and ZsGreen-negative cells, and the sorted cells were mixed with Matrigel and implanted into the flank of female 6-week-old NSG mice at a density of 10^6^ cells per site. Injection sites were monitored weekly for tumor formation. When the tumors reached ~ 3–5 mm, the mouse was treated intraperitoneally with 500 IU/kg rhEpo or vehicle. Three hours after rhEpo treatment, the mice were anesthetized, and cone beam CT images were obtained using an image-guided small animal irradiator. **a** An individualized treatment plan was generated for each tumor to apply a 4 Gy dose to the tumor using a single beam. The smaller green circle represents the target, and the pale green regions represent the regions covered by the applied beam. **b** The mice were administered daily injections of rhEpo or vehicle for a total of 5 days. After the final treatment, the tumors were harvested and digested into single cells. The cell suspensions were analyzed by flow cytometry for the induced BCIC population (StrawberryRed-positive, ZsGreen-positive). The graph represents the normalized %ZsGreen-positive tumor cells observed in at least four animals per group ± SEM

To further explore if this phenomenon occurs also in vivo, we sorted ZsGreen-cODC-negative cells from cells and injected them into NSG mice. Flank tumors were irradiated with a single dose of 0 or 4 Gy (Fig. 3a). Animals were injected with rhEpo once daily for 5 days. When the tumors were explanted on day 6 after irradiation and cells were analyzed by flow cytometry, we found a 1.2-fold increase in the number of induced BCICs in tumors treated with 4 Gy. Treatment of the animals with rhEpo led to a significant 2.3-fold increase in the number of BCICs (Fig. 3b; *p* = 0.02, unpaired, two-sided Student’s *t* test).

### Erythropoietin induces Yamanaka factor expression in breast cancer cells

**Fig. 4:**
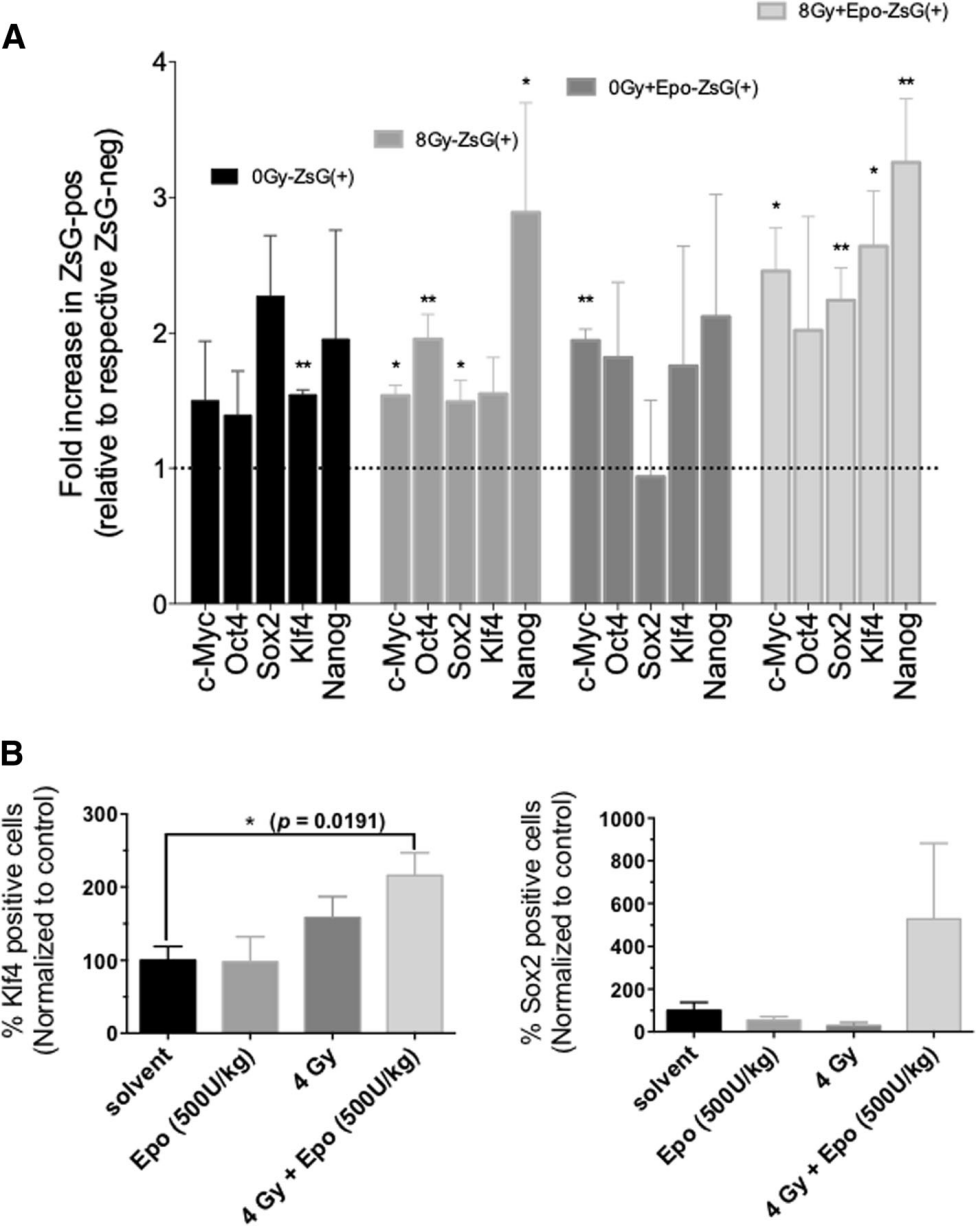
rhEpo induces Yamanaka factor expression in breast cancer cells in vitro and in vivo. SUM159PT-ZsGreen-cODC cells were depleted of intrinsic stem cells by sorting for the ZsGreen-negative population. **a** These cells were plated and, the following day, were treated with 1 IU/ml rhEpo or vehicle followed 1 h later by irradiation with 0 or 8 Gy. Five days later, the cells were sorted for ZsGreen-negative and ZsGreen-positive cells. Expression of the Yamanaka factors were measured by qRT-PCR and normalized to GAPDH and the ZsGreen-negative population from the control sample. Bars represent the average fold increase in mRNA expression obtained from three independent repeats ± SEM. Statistical significance was determined by multiple t tests with a false discovery rate of 1%. * *p* < 0.05, ** *p* < 0.01. **b** BCIC-depleted SUM159PT-ZsGreen-cODC cells were implanted subcutaneously into the flanks of female 6–8-week-old NSG mice. Injection sites were monitored weekly for tumor formation. When the tumors reached ~ 3–5 mm, the mouse was treated intraperitonally with 500 IU/kg rhEpo or vehicle. Three hours after rhEpo treatment, the mice were treated with a 4-Gy dose to the tumor using a single beam. The mice were administered daily injections of rhEpo or vehicle (i.p.) for a total of 5 days. After the final treatment, the tumors were harvested, fixed, embedded, and sectioned. The sections were stained for Klf4 and Sox2, representative images shown. The % stained cells were quantified using a non-biased automated nuclei counting software. The graphs represent the % Klf4- or Sox2-stained cells normalized to the 0 Gy, vehicle-treated group from four individual animals per group ± SEM. Statistical significance was determined by Student’s *t* tests. * *p* < 0.05

We previously reported that radiation led to re-expression of Yamanaka factors in non-BCICs. To test if rhEPo would augment this effect, SUM159PT-ZsGreen-negative cells were obtained by FACS, irradiated with 4 Gy and treated for four consecutive days with 1 IU/ml rhEpo. Five days after irradiation, induced breast cancer initiating cells (iBCICs) were re-sorted by FACS and Yamanaka factor expression was analyzed by qRT-PCR. A single dose of 8 Gy significantly increased c-Myc, Oct4, Sox2, and Nanog mRNA expression in non-irradiated cells while treatment with rhEpo alone significantly increased c-Myc expression (Fig. 4a). Combined treatment with rhEpo and radiation enhanced the effect of radiation leading to a significant increase in c-Myc, Sox2, Klf4, and Nanog expression (Fig. 4a).

Next, we tested if combining irradiation with rhEpo would increase Yamanaka factor protein expression in vivo. Mice, bearing SUM159PT xenografts, were treated with radiation using an image-guided small animal irradiator and rhEpo. Five days after irradiation, tumors were explanted and tumor sections stained for Sox2 and Klf4 and subjected to an automated image analysis. The combined treatment with radiation and rhEpo significantly increased the number of Klf4-positive tumor cells (Fig. 4b; 2.16-fold, *p* = 0.019, unpaired, two-sided Student’s *t* test). The number of Sox2-positive cells after combined treatment increased 5.3-fold but did not reach significance levels (Fig. 4b).

### High serum erythropoietin levels correlate with low numbers of ALDH1A3-expressing breast cancer cells

**Fig. 5:**
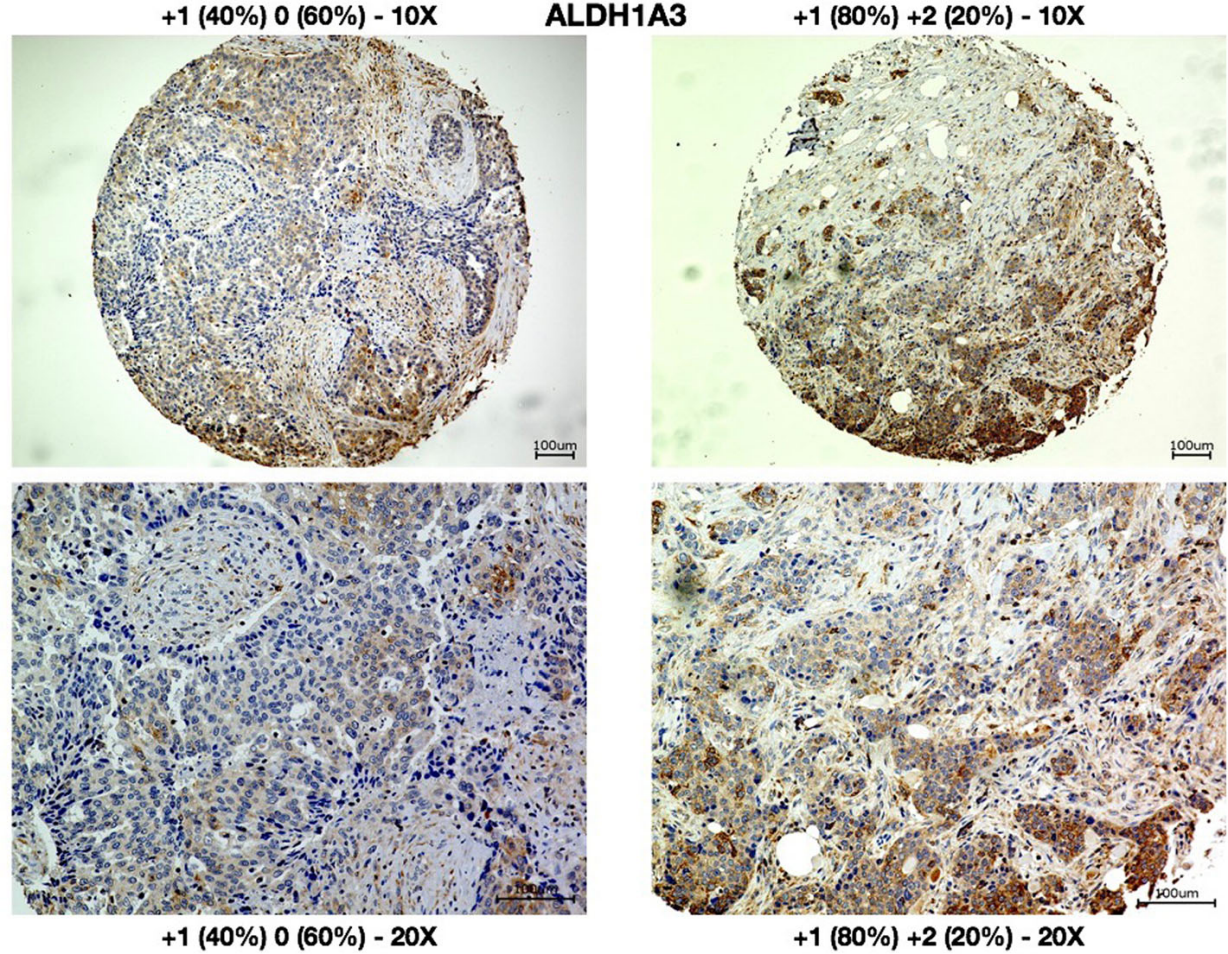
Sample cores of breast cancer specimen stained for ALDH1A3. Sample cores are shown at 10× (upper row) and 25× (lower row). Cores were scored based on the highest staining observed: Core one (left) with 40% of the cells staining “+ 1” and 60% staining “0” was scored “+ 1.” Core two (right) with 80% of the cells staining “+ 1” and 20% staining “+ 2” was scored “+ 2”. Scale bars = 100 μm

Finally, we sought to study if elevated endogenous erythropoietin would increase the number of tumor-initiating cells in breast cancer patients. Ninety-nine patients with primary, non-metastatic breast cancer were included in the study. Patient characteristics are summarized in Table 2. Pre-radiotherapy hemoglobin levels were available for 92 patients. The median hemoglobin concentration was 12.8 g/dl. Serum erythropoietin levels correlated with the disease stage (*p* = 0.049, ANOVA) were elevated (> 19.5 mU/ml) in 29 patients and were inversely correlated with the hemoglobin concentration (*p* < 0.0001). Tumor cores were available for 52 patients. When age, disease stage, receptor status, the presence of a triple-negative tumor, hemoglobin concentration, neoadjuvant chemotherapy, and erythropoietin serum levels were fitted using a logistic regression model, high serum erythropoietin levels correlated with decreased numbers of cells expressing the A3 isoform of aldehyde dehydrogenase 1 (ALDH1A3) (Fig. 5; *p* = 0.0153, Table 3), a marker for BCICs [26–29]. Staining against the proteasome subunit PSMD1, a marker correlated with worsened outcome in glioblastoma [30] and cancer of the head and neck [31], was not informative because all tumor cores showed high or very high expression levels for PSMD1, thus supporting the rare nature of BCICs. Likewise, staining of the tumor sections with and antibody against the A1 isoform of ALDH1 did not show any correlation with serum erythropoietin levels (data not shown).

**Table 2:** Baseline demographic and tumor-related characteristics

Age	Median (range)	60 (29–86)
Race		*No, %*
	African American	6 (6)
	Asian	13 (13)
	Indian	2 (2)
	White	63 (64)
	Other/unknown	5 (5)
BMI	*kg/m* ^*2*^ *, mean (SD)*	
	27.5 (6.0)	
Height	*cm, mean (SD)*	
	162.5 (7.6)	
Weight	*kg, mean (SD)*	
	72.9 (19.9)	
ECOG PS		*No (%)*
	0	85 (86)
	1	6 (6)
	2	5 (5)
	Unknown	3 (3)
Postmenopausal status		*No (%)*
	Yes	69 (70)
Stage	*Stage*	*No (%)*
	0	19 (19)
	I	28 (27)
	II	41 (41)
	III	11 (11)
Neoadj ChT	Yes	20 (20)
Surgery	*Type*	No
	Breast conserving	77
	Mastectomy	11
	Bilat. mastectomy	11
Receptor status		*No (%)*
ER	Positive	77 (78)
	Negative	22 (22)
PR	Positive	66 (67)
	Negative	33 (33)
Her2	Positive	14 (14)
	Negative	70 (71)
	Unknown	15 (15)
Hemoglobin, g/dl		
	Mean (SD)	12.3 (1.7)
	Median (range)	12.8 (8.9–15)
	*Category*	*No (%)*
	< 9	2 (2.2)
	9 to < 10	9 (9.8)
	≥ 10	81 (88)
Erythropoietin, IU/l		
	Mean (SD)	19.2 (9.6)
	Median (range)	16.8 (8.5–60.9)

**Table 3:** Analysis of maximum likelihood estimates

Parameter		DF	Estimate	Standard error	Wald chi-square	Pr > ChiSq
Intercept		1	15.1728	25.4962	0.3541	0.5518
Epo (mIU/mL)		1	− 0.1674	0.0664	6.3653	0.0116
Age		1	− 0.0352	0.0331	1.1282	0.2881
Numeric stage	0	1	− 20.4155	224.3	0.0083	0.9275
Numeric stage	1	1	5.6702	74.7554	0.0058	0.9395
Numeric stage	2	1	5.8172	74.7587	0.0061	0.9380
Hgb		1	− 0.5733	0.3153	3.3058	0.0690
Triple negative	0	1	5.2131	33.6736	0.0240	0.8770
Chemotherapy	0	1	1.0505	0.7933	1.7536	0.1854
Receptor status	0	1	5.6305	33.6720	0.0280	0.8672
Histology	DCIS	1	19.3640	199.3	0.0094	0.9226
Histology	IDC	1	− 9.4229	99.6720	0.0089	0.9247

## Discussion

In general, the structural organization of tumors can be described using two competing models: (1) a stochastic model in which every cell in a tumor has the potential of unlimited self-renewal and multi-lineage potency and (2) a hierarchical model in which this phenotype is restricted to a small number of cancer-initiating cells (CICs) [32]. There is now ample evidence favoring the hierarchical model over the stochastic model in many solid cancers [33–42] including breast cancer [24]. Based on cell surface markers or enzymatic activity of ALDH1, several studies prospectively identified cell populations highly enriched for BCICs [26–29]. Importantly, in clinical breast cancer samples, the expression of the A3 isoform of ALDH1 is predicative of tumor grade, metastasis, and cancer stage [43].

In the present study, we explored if erythropoietin increases the plasticity of breast cancer cells and tested the hypothesis that elevated endogenous serum erythropoietin levels in breast cancer patients increase the aggressiveness of breast cancer through expansion of the pool of BCICs, analogous to the effects seen after treatment of breast cancer cell lines with rhEpo and the unfavorable results from randomized trials using rhEpo in anemic cancer patients [12, 44, 45].

Preclinical studies to date have failed to conclusively describe the effects of rhEpo on tumors, with some studies observing no effect on cancer cells [46], others report anti-cancer activity of rhEpo [47–49], and still others describe tumor-promoting effects of rhEpo [50–55]. We, and others, have reported that radiation induces less ROS formation and DNA DSBs in CICs [21, 30, 56] and that cell cycle checkpoints are activated more efficiently in CICs [57]. This results in a relatively radioresistant phenotype of CSCs [21, 23, 57] and a relative and absolute increase in the number of CICs after fractionated irradiation [22]. However, factoring in the long doubling time of BCICs, which remained unchanged after irradiation, and their intrinsic radiosensitivity, we consistently found absolute numbers of BCICs that could not be easily explained by symmetric division of existing CICs [22]. In a follow-up study, we reported that radiation induced a phenotype conversion of non-tumorigenic breast cancer cells into BCICs [19], thus offering an additional explanation for the observed increase of BCICs after irradiation. In the present study, we confirmed that radiation enriches for putative BCICs in established breast cancer cell lines. Treatment of the cells with rhEpo showed a trend to enhance this phenomenon in vitro. Likewise, removal of BCICs before irradiation confirmed the radiation dose-dependent generation of induced BCICs from non-stem breast cancer cells, an effect that was enhanced by treatment with rhEpo. However, in both experiments, the increase in the number of CICs that could be attributed to rhEpo treatment was rather small. This was in agreement with a previous study finding rhEpo to more efficiently promote breast cancer in vivo than in vitro [58].

We report here for the first time that radiation-induced phenotype conversion can be observed when breast cancer xenografts are irradiated in vivo. This effect was significantly increased when radiation and rhEpo were combined. Given the small effects of rhEpo on radiation-induced phenotype conversion in vitro, it suggests that the effects of rhEpo that lead to increased phenotype conversion in irradiated tumors are at least co-mediated by a stromal component.

As expected, we found an inverse correlation between hemoglobin and serum erythropoietin levels in a cohort of breast cancer patients scheduled to undergo radiation treatment and mean serum erythropoietin levels correlated with disease stage. Henke et al. previously reported a correlation between low serum hemoglobin levels and disease-free survival in early-stage breast cancer patients receiving breast-conserving surgery followed by adjuvant radiotherapy [2].

The data presented in the present study indicate that more advanced breast cancers more efficiently suppress erythropoiesis even in the presence of elevated serum erythropoietin levels. However, when we stained for ALDH1A3, a known marker for breast CICs [43], we found serum erythropoietin levels negatively correlated with the number of putative breast CICs in the tumor sections. This was unexpected and, in the light of our experimental data, suggests different effects of slowly rising endogenous erythropoietin levels in response to tumor-related anemia as opposed to relatively large and rapid increases through application of rhEpo in vitro or in vivo. Another explanation could be a differential effect of endogenous and recombinant human erythropoietin on cancer cells. Endogenous and recombinant erythropoietin differ substantially in their levels of glycosylation and protein structure with consequences for receptor binding affinity and protein stability, differences which are utilized to detect blood doping in athletes [59].

## Conclusions

While all forms of erythropoietin undisputedly stimulate erythropoiesis through the erythropoietin receptor, the consequences of the structural differences for potential off-target effects are currently not well understood and could explain the observed discrepancy between our in vitro and in vivo studies on the one hand and our clinical observations on the other. Further studies are warranted to better understand functional differences of the multiple forms of erythropoietin on tumor cells.

## Additional file

Additional file 1: Primer sequences used in the qRT-PCR. Forward and reverse primers used in real time PCR. (DOCX 12 kb)

## Abbreviations

ALDH1A3: Aldehyde dehydroxygenase 1, isoform A3
BCICs: Breast cancer-initiating cells
bFGF: Fibroblast growth factor 2
CT: Computed tomography
DAB: 3,3′-Diaminobenzidine
DMEM: Dulbecco’s modified Eagle’s medium
EGF: Epidermal growth factor
ELISA: Enzyme-linked immunosorbent assays
FBS: Fetal bovine serum
FDA: Federal Drug Administration
NSG: NOD-*scid* IL2Rgamma^null^
PCR: Polymerase chain reaction
QOL: Quality of life
rhEpo: Recombinant human erythropoietin
RT: Radiation Therapy
