# Supplementary material for: Serum erythropoietin levels, breast cancer and breast cancer-initiating cells
Source: Breast Cancer Res. 2019 Jan 30;21:17. doi: 10.1186/s13058-019-1100-9 (PMC6354373; doi:10.1186/s13058-019-1100-9)
Supplement: Supplementary file 1 — Primer sequences used in the qRT-PCR. Forward and reverse primers used in real time PCR. (DOCX 12 kb) [file 13058_2019_1100_MOESM1_ESM.docx]

**Additional File 1**

**File format: MS Word .docx**

**Primer sequences used in the qRT-PCR**

Forward and reverse primers used in real time PCR

c-MYC-Rev: 5’ cactgtccaacttgaccctcttg 3’

c-MYC-For: 5’ cgtctccacacatcagcacaa 3’

Klf4 –Rev: 5’ ggtccgacctggaaaatgct 3’

Klf4-For: 5’ accaggcactaccgtaaacaca 3’

Oct-4-Rev: 5’ catagtcgctgcttgatcgcttg 3’

Oct-4-For: 5’ gagaaccgagtgagaggcaacc 3’

Sox2-Rev: 5’ ttgcgtgagtgtggatgggattggtg 3’

Sox2-For: 5’ gggaaatgggaggggtgcaaaagagg 3’

Nanog-Rev: 5’ tgcgtcacaccattgctattcttc 3’

Nanog-For: 5’ aatacctcagcctccagcagatg 3’

TBP-Rev: 5’ cacatcacagctccccacca 3’

TBP-For: 5’ tgcacaggagccaagagtgaa 3’

PPIA-Rev: 5’ tctttcactttgccaaacacc 3’

PPIA-For: 5’ atgctggacccaacacaaat 3’

IPO8-Rev: 5’ gaattccacatggtcagagact 3’

IPO8-For: 5’ cgaagttgcggattgcag 3’
